# The Effect of Sodium Nitroprusside Treatment on Storage Ability of Fresh-Cut Potato

**DOI:** 10.3390/foods12010221

**Published:** 2023-01-03

**Authors:** Yukexin Dai, Hong Xie, Xiaoyan Zhao, Yanyan Zheng

**Affiliations:** 1 Beijing Academy of Agriculture and Forestry Sciences, Beijing Key Laboratory of Agricultural Products of Fruits and Vegetables Preservation and Processing, Key Laboratory of Vegetable Postharvest Processing, Institute of Agri-Food Processing and Nutrition, Ministry of Agriculture and Rural Affairs, Beijing 100097, China; 2 College of Food Science, Shenyang Agricultural University, Shenyang 110866, China

**Keywords:** visual quality, biochemical changes, nutritive value, before and after cutting treatment

## Abstract

Quality deterioration is a major problem restricting the fresh-cut potato industry. The present study investigated the effect of sodium nitroprusside (SNP) treatment on the quality of fresh-cut potatoes during short-term storage. The treatment was carried out immediately either before or after cutting, using an SNP concentration of 200 μmol/L. The results showed that SNP treatment inhibited the accumulation of malondialdehyde (MDA) and total soluble solids (TSSs). SNP treatment also decreased the firmness, chewing properties, and ascorbic acid (AsA) content in potatoes, maintaining high levels of total phenols (TPs), total flavonoids (TFs), nitric oxide (NO), and superoxide dismutase (SOD). Furthermore, SNP treatment restrained the rise of phenylalanine ammonia-lyase (PAL), peroxidase (POD), and polyphenol oxidase (PPO), as well as the electrolyte leakage (EL) rate. After SNP treatment, the nitrite content in the potatoes was within security scope. Comparing potatoes treated before and after cutting, the best result was noted in the potatoes soaked in SNP before cutting, which displayed the smallest losses in firmness (11.24%), chewing properties (34.30%), and AsA (40.35%), and maximum increases in TPs (32.84%), TFs (2.83−time), NO (76.11%), and SOD activity (93.15%). Moreover, this group presented the minimum MDA content, EL rate, and TSS values and the lowest PAL, POD, and PPO activities. These results indicated that 200 μmol/L SNP applied for 20 min, particularly before cutting, is an efficient alternative technology that can be used in the fresh-cut potato industry.

## 1. Introduction

Potatoes, one of the four main global crops, have attracted increasing attention from consumers owing to their abundant nutrients, low energy, and high dietary fiber content [1,2]. In recent years, the fresh-cut potato market has boomed due to consumer perceptions of naturalness, convenience, and nutrition [3]. However, fresh-cut products are vulnerable to experiencing serve-quality deterioration, such as decay, softening, browning, nutrient loss, and microbiological spoilage, due to the current fresh-cut processes [4,5]. Such undesirable changes severely restrict the storability and market value of fresh-cut products [6]. To maintain the storage quality of fresh-cut potatoes, considerable studies have been performed, such as those involving ultrasounds [7], heat shock [8], sodium chloride (NaCl) solutions [9], and ascorbic acid (AsA) [10].

As a free radical gas, nitric oxide (NO) plays an essential role in preserving the quality of stored fruits [11,12]. A study of fresh-cut peaches indicated that application of a 10 μmol/L NO aqueous solution for 5 min effectively enhanced the activities of superoxide dismutase (SOD) and catalase (CAT); inhibited the browning activities of phenylalanine ammonia-lyase (PAL), polyphenol oxidase (PPO), and peroxidase (POD); altered the total phenols (TP); and decreased the malondialdehyde (MDA) content, thus extending the shelf life of fresh-cut peaches [13]. As a donor of NO gas, a solution of sodium nitroprusside (SNP) mainly consisting of Fe^2+^, nitric oxide (NO), and five cyanide anions, which have ability to react with cells in plants, releases NO gas into a water solution. It has been reported that, compared with control, 200 μmol/L SNP treatment has the potential to preserve fruit quality by enhancing mango firmness, delaying the development of undesirable color, and decreasing weight loss, as well as titratable acid (TA), total soluble solid (TSS), and MDA accumulation [14]. Research has shown that immersing button mushrooms in 15 μmol/L SNP solution for 3 min can preserve their hard firmness and uniformity in color and maintain the content of 1-octen-3-ol, which is related to button mushroom flavor [15]. The abovementioned studies supply valuable evidence that the application of SNP can exert a positive effect in maintaining the storage quality of fruits and vegetables.

Although the utilization of SNP is a safe and low-cost technology with high operability, it is rarely applied to fresh-cut products, especially in the fresh-cut potato industry. Thus, the aim of this study is to discuss how the quality of fresh-cut potatoes is affected by soaking in SNP before and after cutting.

## 2. Materials and Methods

### 2.1. Plant Materials and Treatment

Potatoes (*Solanum tuberosum* L. cv Netherlands 15) were purchased from a vegetable market in Shandong, China. Scatheless potatoes with a uniform mass (400–450 g) and length (9–12 cm) were selected. The potatoes were washed with clean water two times, peeled, and cubed. Potatoes were divided into three groups, with approximately 4000 g of potatoes in each group. Group A was immersed in deionized water for 20 min as a control (CK), the potato cubes in group B were immersed in 200 μmol/L of SNP solution for 20 min then cut into 5 mm slices (SNP-soaking before cutting), and the potato cubes in group C were first cut into 5 mm slices and then immersed in 200 μmol/L of SNP solution for 20 min (SNP-soaking after cutting). A fruit-exclusive centrifuge (YZ-600, Guangzhou, China) was used to remove the potato surface water, followed by vacuum packaging (DZ-1000, Shenzhen, China) and storage at 4 ± 0.5 °C, with 90%–95% humidity for 8 days. Three bags (each weighing 250 g) were sampled at 2-day intervals. Four slices of potato were used for quality assessment of appearance, color, firmness, electrolyte leakage (EL), and TSSs. The other two bags of potatoes were frozen with liquid nitrogen and stored at −80 °C for measurement of the remaining indices. The experiments were conducted three times (*n* = 3), unless specifically stated in the article.

### 2.2. Visual Appearance and Color

The surface color was measured with a chromameter (CM-700 d, Konica Minolta Co., Osaka, Japan). The indices L*/(lightness), a*/(red–green) and b*/(yellow–blue) were used to represent the color change in the fresh-cut potato slices. The chromatic aberration ΔE* represents the change in color of the sample.

$$\Delta E^{*}=\sqrt{{\left(L_{0}^{*}-L_{X}^{*}\right)}^{2}+{\left(a_{0}^{*}-a_{X}^{*}\right)}^{2}+{\left(b_{0}^{*}-b_{X}^{*}\right)}^{2}}$$

where *L*_0_*, *a*_0_*, and *b*_0_* denote the tristimulus color on day 0 and L_x_*, a_x_*, and b_x_* denote the tristimulus color on the indicated day of sampling [1]. Changes in the visual appearance of fresh-cut potato were monitored with a camera.

### 2.3. Total Soluble Solids (TSSs)

Potatoes were crushed and homogenized to determine the TSS content. TSSs were measured with a refractometer (PAL-1, Atago, Tokyo, Japan).

### 2.4. Texture

Texture analysis was performed using a texture analyzer (TA-XT plus, Stable Micro Systems, Surrey, UK), equipped with a 5 mm diameter cylindrical probe. The basic parameter settings were as follows: pretest speed of 1 mm/s, test speed of 1 mm/s, post-test speed of 10 mm/s, distance of 7 mm, and trigger force of 5 g [10]. The textural parameters were firmness (N) and chewing work (mJ).

### 2.5. Electrolyte Leakage (EL) Rate

The EL was determined with a conductivity meter (DDS-12A type, Shanghai, China). Thirty holes from five potato slices were immersed in 35 mL of distilled water. Subsequently, the initial electrolyte leakage (EI_0_) was determined after 1 h [16]. The ultimate electrolyte leakage (EI_1_) was assayed after heating at 100 °C for 10 min and cooling down to room temperature. The electrical conductivity is represented as EI_0_/EI_1_ × 100%.

### 2.6. Enzyme Activity

PAL activity was measured with a phenylalanine ammonia-lyase activity assay kit (BC0210, Solarbio, Beijing, China), with reference to Li et al. [17]. The reaction system was prepared with 20 μL of crude enzyme solution, 780 μL of boric acid buffer (pH 8.8, 50 mmol/L), and 200 μL of L-phenylalanine solution (20 mmol/L). The mixture was incubated at 37 °C for 30 min. Absorbance was -read at 290 nm. A change in absorbance at 290 nm of 0.1/min was defined as unit enzyme activity. PAL activity was expressed in U/g of fresh tissue weight (U/g FW).

POD activity was measured using a peroxidase activity test kit (BC0090, Solarbio, Beijing, China), according to the manufacturer’s protocol. The measurement method was as follows. Firstly, 3 mL of ice-cold acetate buffer was used to extract peroxidase from frozen potato issue. Then, 520 μL of acetate buffer (pH 5.5, 50 mmol/L), 130 μL of 0.5 mol/L H_2_O_2_ solution (0.5 mol/L), 135 μL of guaiacol solution (25 mol/L), 270 μL of distilled water, and 15 μL of supernatant were added, successively, into EP tubes and mixed. Finally, 700 μL of the reaction was immediately transferred into 1 mL glass cuvettes. Absorbance was read at 470 nm. One unit of POD activity was equal to the increase in absorbance of 0.1 per min. The POD activity was expressed as U/g of fresh tissue weight (U/g FW).

PPO activity was determined using a commercial kit (BC0190, Solarbio, Beijing, China), according to a previous method with minor modification [17]. Briefly, 3 mL of ice-cold acetate buffer was used to extract 3 g of potato supernatant. The reaction system of the control and assay cube includes 100 μL of supernatant, 40 μL of acetic acid–sodium acetate buffer (pH 5.5, 50 mmol/L), and 1 mL catechol (0.05 mol/L). The supernatant of the control cube needs to be boiled with a 100 °C water bath for 10 min. Absorbance was read at 410 nm. PPO activity was expressed in U/g of fresh tissue weight (U/g FW).

### 2.7. Superoxide Dismutase (SOD) Activity

The activity of SOD was assayed with a spectrophotometer, using a commercial kit (BC0170, Solarbio, Beijing, China), following manufacturer’s instructions. Firstly, 3 g of frozen issue was weighed, and 1 mL of phosphate buffer (pH 7.8, 0.5 mol/L) was added for crude enzyme solution extraction. Then, 240 μL of 1 EDTA-Na2 solution (100 μmol/L), 60 μL of nitroblue tetrazolium solution (750 μmol/L), 180 μL of xanthine oxidase, 30 μL of methionine solution (130 mmol/L), and 90 μL of crude enzyme solution were added to a test tube. It is worth noting that, for the control tube, the crude enzyme solution was instead with distilled water. The mixture was incubated at room temperature for 30 min, and absorbance was read at 560 nm. SOD activity was expressed in U/g of fresh tissue weight (U/g FW).

### 2.8. Ascorbic Acid (AsA) Content

AsA content was measured with an ascorbic acid activity test kit (BC1230, Beijing Solarbio, Beijing, China). Two grams of potato-sample extract was blended with 2 mL of extracting solution. After being centrifuged (8000 r/min), for the determination tube, 0.1 mL of extract was mixed with 0.9 mL of intricate solution, consisting of 0.5 mL metaphosphoric acid–acetic acid solution (0.1 g/mL), 0.1 mL of 5% sulfuric acid, and 0.3 mL of ammonium molybdate solution (0.05 g/mL). The standard tube used 100 μL of standard liquids instead of sample. After mixing, then, both measurement and standard tubes accurately transferred 1 mL to a quartz colorimetric utensil. Absorbance was read at 265 nm.AsA activity was expressed in nmol/g of fresh tissue weight (nmol/g FW).

### 2.9. Malondialdehyde (MDA) Content

One gram of frozen potato sample was placed into a 10 mL centrifuge tube with 5 mL of trichloroacetic acid solution (5% *w*/*v*). The samples were then centrifuged at 8000 r/min. The MDA reaction included 2 mL of trichloroacetic acid solution (0.6% *w*/*v*) and 2 mL of supernatant, which were heated at 100 °C for 10 min [18]. After heating, the mixed solution was cooled to room temperature, and the absorbance at 450 nm, 532 nm, and 600 nm was measured.

### 2.10. Nitric Oxide (NO) Content

Two grams of frozen potato was accurately weighed into a sample in a 10 mL tube with 2 mL of extraction solution. After being centrifugated (4 °C 8000 r/min), the supernatant was collected. The NO content was determined according to the instructions of a commercial reagent kit (BC1470, Beijing Solarbio, Beijing, China). The absorbance was measured at 550 nm. The content was expressed as μmol/kg of fresh tissue weight (μmol/kg FW).

### 2.11. Total Phenols (TPs) and Total Flavonoids (TFs)

The TPs in the fresh-cut potatoes was determined using the Folin–Ciocalteu reagent method [19]. An amount of 3 g of frozen sample was weighed, ground with 25 mL of methanol, and centrifuged (4 °C 8000 r/min). The reaction system included 1 mL of supernatant, 1 mL of Folin–Ciocalteu reagent, and 2 mL of saturated Na_2_CO_3_ solution. It should be emphasized that the reaction needed to be carried out in the dark. The absorption was measured at 765 nm.

The calibration curve was performed by using gallic acid at concentrations of 0–50 mg/L with the regression coefficient R^2^ = 0.9932 and equation y = 0.039x + 0.0814. The total phenolic content was expressed in terms of g/kg of fresh tissue weight (g/kg FW).

TF measurements were recorded according to Prima et al. [20]. One milliliter of supernatant was mixed with 0.3 mL of 5% sodium nitrite solution and 4.0 mL of distilled water. Afterward, 0.3 mL of 10% aluminum chloride solution and 20 mL of 1% sodium hydroxide were added to the tube, which was incubated at room temperature for 10 min. The absorbance was measured at 510 nm.

### 2.12. Nitrite Content

Extraction and further determination of nitrite content were performed according to the International Organization for Standardization standard [21]. Briefly, a mixture of 15 mL of saturated borax, 5 mL of zinc acetate, and 5 mL of potassium ferricyanide was used to extract nitrite from 5 g of frozen potato tissue. A solution of p- naphthalenediamine acid and naphthylenediamine hydrochloride was used to generate a standard curve and calculate the nitrite content. The nitrite content in the samples was presented as mg/kg.

### 2.13. Analysis of Data

IBM SPSS 27 and Origin 2011 were used for statistical analysis. The error bars represent the SDs of the means in all graphs. Different letters at the same storage time in the same figure denote significant differences (*p* < 0.05).

## 3. Results and Discussion

### 3.1. Effect of Treatment with SNP on Potato Visual Appearance and Color

**Figure 1:**
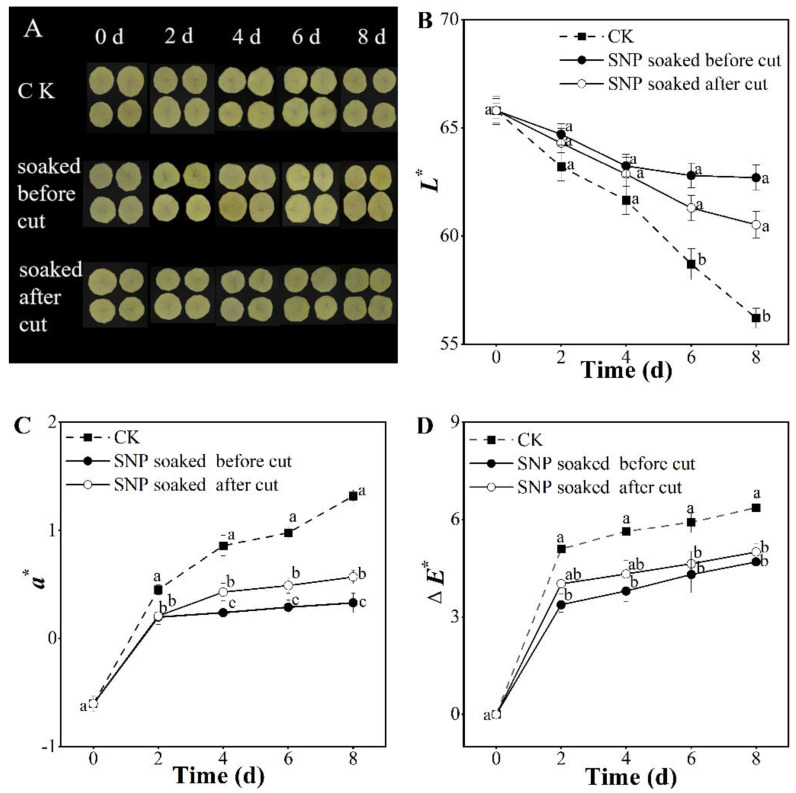
Effect of SNP treatment on visual appearance (**A**) and color of L* (**B**), a* (**C**), and ΔE* (**D**) of fresh-cut potatoes. Different letters indicate significant differences among treatments at same storage time (*p* < 0.05).

Visual appearance is an essential aesthetic quality [22]. No unacceptable surface appearance features were observed in the potato slices treated with SNP and those in the control group after eight days (Figure 1A), which indicated that the above treatment maintained the visual quality of fresh-cut potatoes for eight days. To further quantify the visual differences between these groups, the color parameters (L*, a*, and ΔE* values) were investigated, as shown in Figure 1B–D. In the SNP groups, the a* and ΔE* values showed lowered values of 0.75–0.99 and increased values of 1.37–1.67, respectively, while L* was gradually boosted to 7.67–11.54% on eighth day compared to control. Among the three treatments, the potato treated with SNP before cutting showed the best color during short-term storage.

There was no difference in the visual inspection between the SNP and control groups after eight days (Figure 1A). However, the color results indicated that the potatoes in the SNP group had a better surface color than those in the control group, confirming results previously obtained in peaches [23] and Hami melons [24]. Among the treatment groups here, SNP-soaking before cutting showed superior color maintenance compared with SNP-soaking after cutting.

### 3.2. Effect of Treatment with SNP on Potato Texture

**Figure 2:**
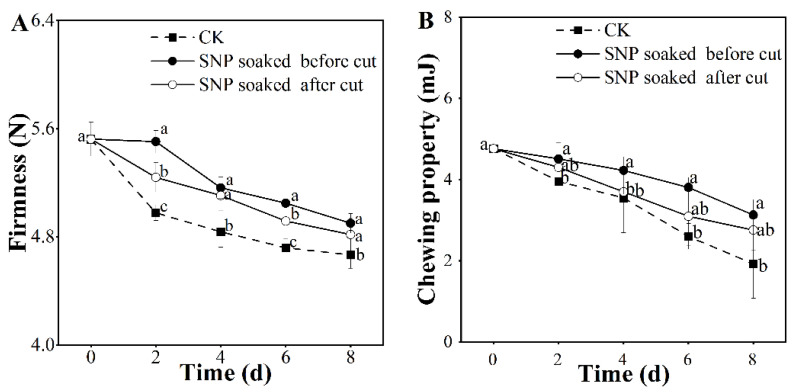
Effect of SNP treatment on texture of fresh-cut potatoes. Firmness (**A**) and chewing properties (**B**). Different letters indicate significant differences among treatments at same storage time (*p* < 0.05).

Texture is a key factor when evaluating fresh-cut potato commodities [3]. In a recent study, the texture of fresh-cut potato slices was evaluated by assessing the firmness and chewing properties (Figure 2). The firmness data displayed a sequential descent in both SNP and control groups; however, SNP treatment produced a descent delay in hardness (Figure 2A). Moreover, potatoes that received SNP treatment before cutting exhibited higher hardness values during storage. The effect of SNP treatment on the chewing properties of potatoes is shown in Figure 2B, in which the trend in variation coincided with the trend in firmness (Figure 2A). We found that the chewing value of potatoes treated with SNP before cutting was much higher than that of the other treated samples during the entire storage period, in which 34.30%, 42.20%, and 59.53% chewing loss was found in potatoes immersed in SNP before cutting, those immersed in SNP after cutting, and the control group, respectively, from day zero to the eighth day.

Studies have shown that SNP maintains firmness in blueberries [14] and tomatoes [25] by reducing the degradation of the cell wall. In our study (Figure 2), SNP successfully delayed the decreases in firmness and chewing work. Thus, soaking in SNP before cutting was found to be an efficient application method to preserve the hard texture of potatoes.

### 3.3. Effect of Treatment with SNP on Potato Membrane Integrity

**Figure 3:**
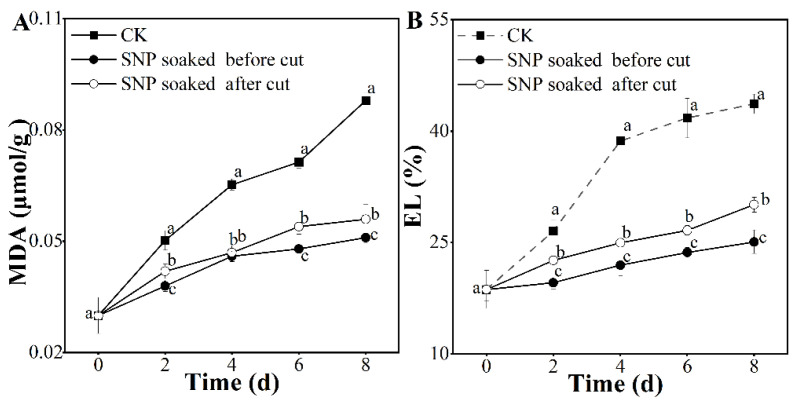
Effect of SNP treatment on membrane integrity of fresh-cut potatoes. MDA (**A**) and EL (**B**). Different letters indicate significant differences among treatments at same storage time (*p* < 0.05).

Lipid oxidation leads to damage to the cell membrane, resulting in further contact between the enzyme and browning substrate, which eventually accelerates unacceptable browning in fresh-cut potatoes [11,26]. As the ultimate product of membrane lipid peroxidation, the MDA content increased continuously (Figure 3A). However, the increase in MDA content in SNP–treated potatoes was relatively slower than that in the control. Moreover, in this study, we observed 0.7−–fold, 0.86−–fold, and 1.93−–fold increases in MDA contents in potatoes treated with SNP before cutting, those treated with SNP after cutting, and those in the control group, respectively, on the eighth day compared with the initial value. During storage, the group of potatoes soaked in SNP before cutting had a significantly lower MDA content than the other groups (*p* < 0.05). The above data showed that SNP-soaking before cutting minimizes oxidative injury in fresh-cut potatoes during storage. The EL showed a continuous increasing trend during storage (Figure 3B). Notably, the rate at which the EL rose in potatoes treated with SNP was lower than that in the control group of potatoes, indicating that SNP treatment can retard the rise of EL. At the end of storage, the EL in the untreated group was approximately 43.68%; however, potatoes treated with SNP-soaking before cutting and SNP-soaking after cutting presented EL values of only 25.10% and 30.10%, respectively. The above data suggest that SNP treatment before cutting can minimize the increase in EL, displaying better maintenance of the intact cell membrane structure [27].

In summary, SNP treatment before cutting significantly delayed the increases in MDA and EL (*p* < 0.05) in this study (Figure 3), which are related to minor cell wall damage [28].

### 3.4. Effect of Treatment with SNP on Potato Nutritional Properties

**Figure 4:**
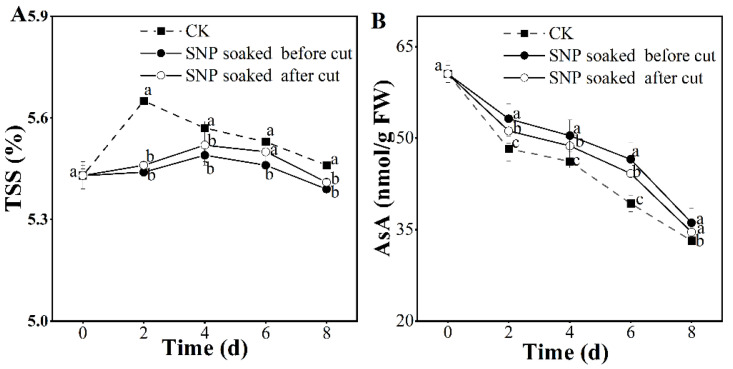
Effect of SNP treatment on nutritional properties of fresh-cut potatoes. TSSs (**A**) and AsA (**B**). Different letters indicate significant differences among treatments at same storage time (*p* < 0.05).

TSSs can be viewed as an index that reflects the maturity and nutritional quality of fresh-cut fruits. In this study, the TSS content displayed a tendency to increase and then decrease (Figure 4A). The TSS content rises during ripening and falls after full ripeness is achieved [29]. The TSS value in untreated potatoes peaked on the second day, while that in the SNP group showed the maximum on the fourth day, indicating that SNP treatment delayed the accumulation of TSSs, which is in agreement with Ren et al. [30]. Moreover, during storage, we observed that the group of SNP soaked before cut had the lowest TSS value, indicating that this treatment can retard the accumulation of TSSs. The excessive increase in TSSs is correlated with the disintegration of polymeric carbohydrates [31]. Processing potatoes by immersion in SNP before cutting reduces the breakdown of polymeric carbohydrates, thereby alleviating the accumulation of TSSs. AsA is not only an essential nutrient but also an antioxidant that relieves stress caused by ROS, thus exerting a positive influence to alleviate potato browning [32]. There was a linear decrease in the AsA content during storage (Figure 4B), which was postponed by the SNP treatments, indicating that SNP maintained the AsA content. During storage, potatoes receiving SNP treatment before cutting retained a higher AsA content, suggesting that this treatment preserved the AsA content in fresh-cut potatoes.

With the extension of storage time, fresh-cut fruits may be confronted with problems involving the loss of nutritional substances [32,33,34]. In this study, both SNP treatments reduced the loss in AsA content and lessened the increase in TSSs to some degree (Figure 4), which is accordance with Ma et al. [9]. Furthermore, potatoes soaked in SNP before cutting presented the minimum TSS content and maximum AsA retention. The above data indicate that SNP–soaking before cutting is beneficial for maintaining the nutritional substances in potatoes and alleviating potato maturity.

### 3.5. Effect of Treatment with SNP on Potato Antioxidant Properties

**Figure 5:**
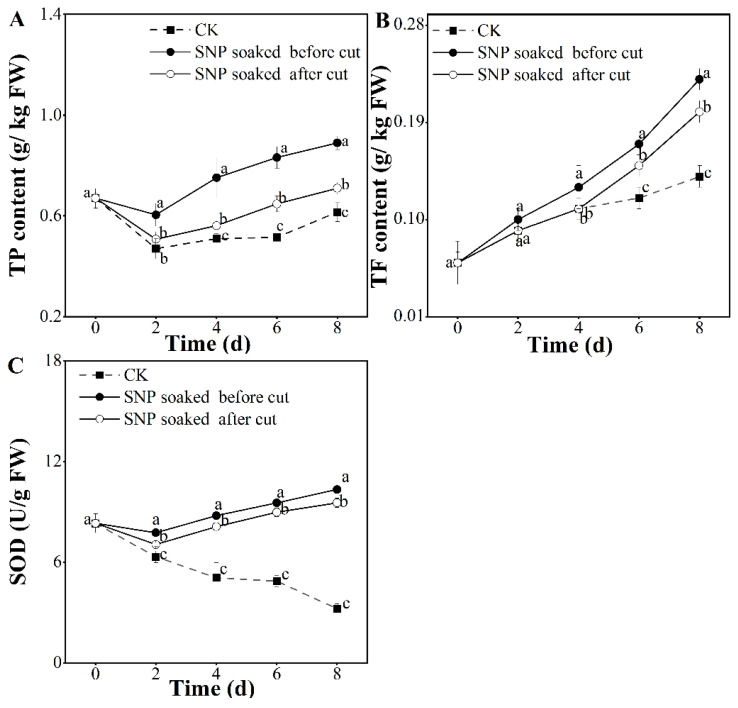
Effect of SNP treatment on antioxidant properties of fresh-cut potatoes. TP (**A**) and TF content (**B**) and SOD activity (**C**). Different letters indicate significant differences among treatments at same storage time (*p* < 0.05).

The effect of TP content is illustrated in Figure 5A. The results showed that the TP content decreased slightly on the second day and subsequently increased in both the SNP and control groups. During storage, potatoes treated with SNP before cutting presented significantly higher TP contents than the other groups during storage (*p* < 0.05). A previous study corroborated that a lower TP content is related to phenolic oxidation [16]. However, in the present study, the highest TP content was observed in potatoes treated with SNP before cutting, which indicated that SNP prohibited phenolic oxidation [34,35]. The TF content increased gradually with storage duration (Figure 5B). This retention of TFs was more prominent in the SNP group, which is similar to research on bananas [36] and grapes [37]. On the final day of this study, it was explicitly noted that potatoes receiving SNP treatment before cutting had the maximum TFs (0.23 g/kg) on the eighth day, while this value was the lowest in the control group (0.14 g/kg) after the same storge time. Moreover, we observed that potatoes soaked in SNP before being cut maintained the highest TF level (*p* < 0.05), which is probably due to the higher enzyme activities of POD and PAL [38]. The variations in SOD activity in each group were inconsistent. In the SNP group, a clear increment was observed, whereas in the control group, a tendency to increase and decrease during storage time was seen (Figure 5C). The data also showed that on the eighth day, the loss in SOD activity in the control group was 9.76%; however, potatoes immersed in SNP displayed a boost to 69.08%–93.15%. Among these groups, SNP-soaking before cutting produced significantly higher SOD activity than the other groups (*p* < 0.05), indicating that this treatment better maintained SOD activity.

In this study, the TP and TF contents and SOD activity were selected to evaluate the antioxidant activity. The data in Figure 5 show that the potatoes that were treated with SNP before cutting had significantly enhanced TP and TF contents and SOD activity during storage (*p* < 0.05), which were related to their high antibrowning ability and ROS scavenging capacity [39].

### 3.6. Effect of Treatment with SNP on Potato Antibrowning Ability

**Figure 6:**
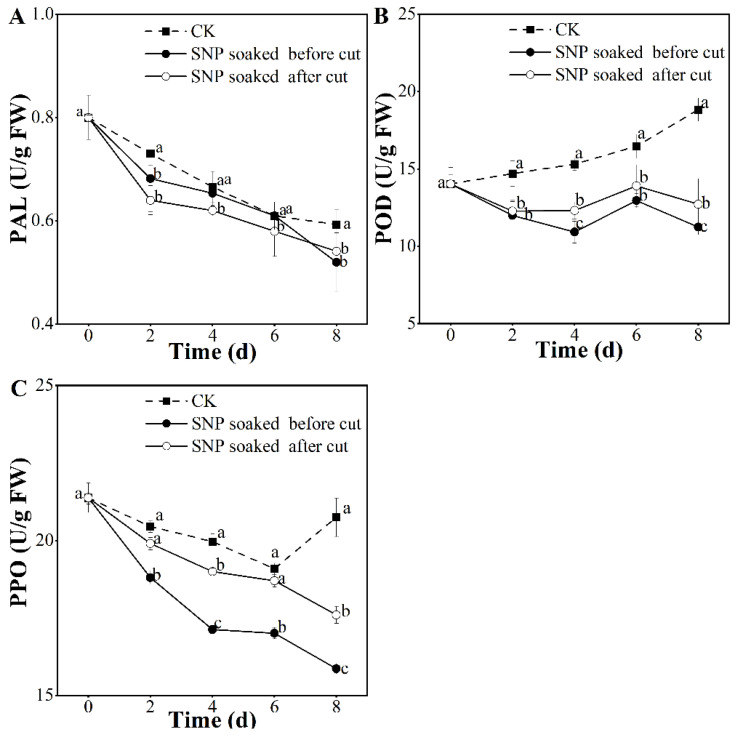
Effect of SNP treatment on enzyme activities PAL (**A**), POD (**B**), and PPO (**C**) of fresh-cut potatoes. Different letters indicate significant differences among treatments at same storage time (*p* < 0.05).

The activity of PAL, which participates in phenylpropanoid metabolism [40], showed a constant decrease during storage (Figure 6A). On the eight day, the PAL activity in potatoes treated with the SNP solution was 0.88–0.91 times that of the control, indicating that SNP lowered PAL activity, which is consistent with a study on peaches [13]. Browning of fresh-cut potatoes is related to the enzymes PPO and POD. In this study, different groups presented different variations in POD activity (Figure 6B). After the SNP treatments, we observed an increasing tendency from the zeroth to the sixth day, which then diminished until the end of the experiment. In the control group, the POD activity showed successive growth. On the last (eighth) day, the POD activities in the control, SNP-treated before cutting, and SNP-treated after cutting groups were 18.83 U/g, 11.25 U/g, and 12.73 U/g, respectively, indicating that SNP-soaking before cutting was the optimal treatment to restrain POD activity. PPO activity presented a tendency to decrease from day zero to the sixth day. Then, with the extension of the storage time, POD activity in the control group increased, while that in the SNP group continuously decreased (Figure 6C). Notably, potatoes treated with SNP before cutting displayed significantly restrained PPO activity (*p* < 0.05) during storage at 4 °C, which signified that this treatment has a powerful ability to inhibit the enzyme PPO. This restraint on PPO enzyme activity is related to the ability of SNP to react with the active center of PPO to form a copper–nitrosyl complex (NO-Cu-PPO) [41].

In summary, considerable antibrowning effects were observed after SNP treatment, which is consistent with research on the winter jujube [42]. Among the groups in this study, potatoes treated by soaking in SNP before cutting had the optimal potential to minimize browning-related enzymes (Figure 6). A previous study confirmed that browning is highly correlated with a reduction in membrane permeability, which causes an unacceptable brown color [10]. In this study, SNP application before cutting maintained a complete cell membrane structure (Figure 3), thereby inhibiting the browning enzymes and preserving the original acceptable surface color of the potatoes (Figure 1B–D).

### 3.7. Effect of Treatment with SNP on Potato NO Content

**Figure 7:**
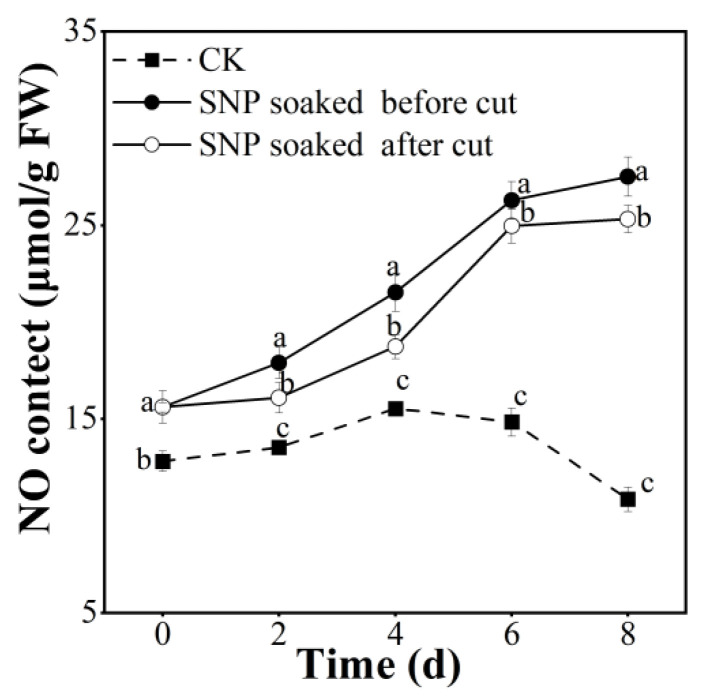
Effect of SNP treatment on NO content of fresh-cut potatoes. Different letters indicate significant differences among treatments at same storage time (*p* < 0.05).

NO is a considerable donor, which is beneficial to maintain fruit storage quality. In this study, the NO content in potatoes treated with or without SNP increased from day zero to the fourth day, after which the potatoes in the SNP group displayed a continuous rise, while those in the control group showed an unfortunate decrease. It is clearly displayed in Figure 7 that the NO content in SNP groups was higher than that in the control group. Among the SNP groups, treatment before cutting produced results that were significantly superior to those of the other groups (*p* < 0.05). These NO data indicated that SNP enhanced the NO content, and SNP treatment before cutting was better. Similarly, treating blueberries with 0.1 mmol/L SNP for 10 min can boost the NO content to 3–5 μmol/g [14], which is consistent with our current study viewpoint.

### 3.8. Effect of Treatment with SNP on Potato Edible Safety

**Figure 8:**
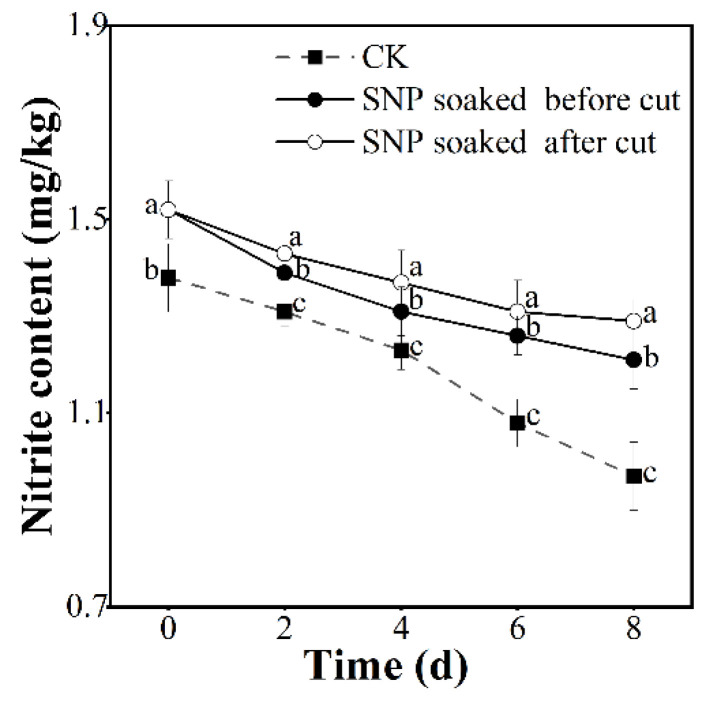
Effect of SNP treatment on nitrite content of fresh-cut potatoes. Different letters indicate significant differences among treatments at same storage time (*p* < 0.05).

In this study, the nitrite content was used to evaluate the edible safety of fresh-cut potatoes treated with SNP. With the extension of storage time, the nitrite content in the fresh-cut potatoes treated with SNP before being cut was significantly lower than that in the potatoes treated with SNP after being cut (Figure 8). Additionally, the nitrite content in the untreated group was significantly lower than that in the remaining groups (*p* < 0.05), suggesting that SNP treatment induced nitrite accumulation slightly. However, it was noted in JECFA 2002 [43] that the human acceptable daily intake (ADI) of nitrite is 0–0.07 mg/kg. This guideline indicated that the treatment applied in our study (immersing potatoes in 200 μmol/L SNP for 20 min) can be applied as a safe preservation technology in the fresh-cut potato industry.

## 4. Conclusions

Fresh-cut potatoes are an indispensable food for dining tables. However, how to better preserve their quality is a challenge. In this study, we found that soaking potatoes in 200 μmol/L SNP for 20 min before cutting delayed undesirable changes in quality. This treatment delayed the degradation of texture, nutrients, and surface color, minimized the damage from lipid oxidation, enhanced the antioxidant activity, and inhibited browning-related enzymes. Moreover, the nitrite data showed that treatment with 200 μmol/L SNP for 20 min gave results within the permissible ADI range. Thus, soaking potatoes in 200 μmol/L SNP for 20 min before cutting would be a promising application for preservation in the fresh-cut potato industry.

## Data Availability

Data are contained within the article.
